# Pediatric ACEs and related life event screener (PEARLS) latent domains and child health in a safety-net primary care practice

**DOI:** 10.1186/s12887-023-04163-2

**Published:** 2023-07-17

**Authors:** Morgan Ye, Danielle Hessler, Derek Ford, Mindy Benson, Kadiatou Koita, Monica Bucci, Dayna Long, Nadine Burke Harris, Neeta Thakur

**Affiliations:** 1 San Francisco Departments of Medicine and Epidemiology and Biostatistics, University of California, 500 Parnassus Avenue, 94143-0841 San Francisco, CA USA; 2 San Francisco Department of Family and Community Medicine, University of California, 500 Parnassus Avenue, 94143 San Francisco, CA USA; 3 Center for Psychological and Social Health, LLC, 2918 Westover Dr, Danville, VA 24541 USA; 4 San Francisco Department of Pediatrics, University of California, 5220 Claremont Ave, Oakland, CA 94609 USA; 5 Center for Youth Wellness, 3450 3rd St, 94124 San Francisco, CA USA

**Keywords:** ACEs, Adverse childhood experiences, PEARLS, Factor analysis, Child abuse

## Abstract

**Background:**

Research examining the connections between individual adverse childhood experiences (ACEs) and how groupings of interrelated adversities are linked with subsequent health is scarce, limiting our understanding of risk during a period of rapid expansion of ACE screening in clinical practice. The study objective was to conduct a psychometric analysis to derive latent domains of ACEs and related life events and assess the association between each domain and health outcome.

**Methods:**

Participants (3 months-11 years) were recruited from the University of California San Francisco Benioff’s Children Hospital Oakland Primary Care Clinic. Children were screened with the Pediatric ACEs and Related Life Events Screener (PEARLS) (n = 340), which assessed 17 total ACEs and related life events, including forms of abuse, household challenges, and social risks. Domains were constructed using confirmatory factor analysis and associations between the three identified domains and 14 health outcomes were assessed using multivariable linear and logistic regression models.

**Results:**

Three PEARLS domains were identified: *Maltreatment* (ω = 0.73, ɑ=0.87), *Household Challenges* (ω = 0.70, ɑ=0.82), and *Social Context* (ω = 0.55, ɑ=0.70). Measurement invariance was supported across both gender and screening format. All domains were associated with poorer general and behavioral health and stomachaches. *Maltreatment* and *Social Context* were additionally associated with eczema while only *Social Context* was associated with increased odds of reporting headaches and somatic symptoms.

**Conclusion:**

In an underserved, urban west-coast pediatric population, the PEARLS found three adversity domains of *Maltreatment, Household Challenges*, and *Social Context* that all had an independent statistically significant association with poorer child health. The results provide a timely and more nuanced representation of risk that can inform clinical practice and policy using more targeted resources and interventions.

**Supplementary Information:**

The online version contains supplementary material available at 10.1186/s12887-023-04163-2.

## Background

Studies have consistently shown that adverse childhood experiences (ACEs) are associated with chronic conditions and proximal poorer health outcomes in childhood/adolescence and later in adulthood [1–3]. Racial/ethnic and socioeconomic disparities exist in ACEs exposure, where Black, Hispanic, and multiracial individuals and those with lower income and education are disproportionately burdened with ACEs [4]. The toxic stress response, which includes neuro-endocrine-immune and genetic regulatory alterations, is considered an important mechanism of how cumulative exposures to early adversities increase risk of morbidity and mortality throughout the life course [5–7]. Cumulative lifetime exposure to social risk factors, such as poverty, discrimination, and unsafe neighborhoods, may trigger similar pathways to toxic stress as traditional ACEs [8]; thus, explicitly including these measures into adversity constructs can have meaningful impact on screening and intervention, especially in safety-net systems that care for underserved populations. Although screening for risk factors for toxic stress has been recommended by the American Academy of Pediatrics [7], screening is only now being more widely incorporated into primary care practice [9].

ACE assessments and related stressful life events have been traditionally implemented and applied as either single adversities or as a cumulative ACE score (i.e., numeric count of total adversities experienced), the latter of which is a powerful tool in assessing risk of toxic stress [2]. However, complementary work examining empirical groupings of individual ACEs (e.g., domains or areas of adversity) and their impact on health remains scarce. Among studies that have examined for domains in assessments of childhood adversity, most have focused solely on the traditional ACE items of maltreatment and household challenges [10–14]. Few have incorporated a wider range of social factors and only examined measurement invariance across sociodemographic characteristics [15–18]. When confirmatory factor analysis is used (i.e., confirming construct validity of factors), measurement invariance across factors can be established if the same questionnaire used in different groups, e.g., gender, shows the factors operate in the same way in each group [19]. As screening for ACEs in primary care settings increases, the urgency to understand the inter-connections between both traditional ACEs and broader social factors, as well as their relationships with health, has been amplified. For example, determining adversity domains may uncover patterns and co-occurrence of ACEs/social risks and mechanisms through which specific combinations of adversities might impact health outcomes [20]. Thus, validation of adversity domains in actively used screening tools, such as the Pediatric ACEs and Related Life Events Screener (PEARLS) [21], has immediate potential impacts on intervention programs and policy aimed at preventing poor health outcomes. Alongside the cumulative ACE score approach – focused on cumulative risk, the identification and application of adversity domains (i.e., how different adversities group together) provides an additional opportunity to more precisely target interventions, strengthen linkages to referrals and resources for patients, and support clinicians in implementing more appropriate referrals.

The Pediatric ACEs and Related Life Event Screener (PEARLS) was developed to measure both ACEs and related life events, including multiple social risk factors [21]. Previous research using this screener has documented that both lifetime exposure to traditional ACEs and to common social risks have a similar and negative cumulative risk to child health [22]. No psychometric properties have been developed for PEARLS so far. To extend these findings and support application and alignment of the PEARLS, toxic stress mitigation, and resource linkage in primary care, the aims of the current study were to: (1) conduct a psychometric analysis to derive a set of latent domains from the PEARLS; (2) demonstrate that the domains maintain measurement invariance across key factors for pediatric care (e.g., age groups, gender, and screening format); and (3) assess the association between domains and pediatric clinical, mental, and behavioral outcomes.

## Methods

### Study population and design

We used data from the Pediatric ACEs and Resiliency Study, a randomized control study designed to: (1) examine the relationship between ACEs and health outcomes in children and caregivers over time, (2) validate an ACEs screening in a pediatric health care setting, and (3) assess preventative interventions for children with or at risk for toxic stress. Primary results and details of the study design have been presented elsewhere [21, 22]. Briefly, from March 2017 to October 2018, 555 participants ages 3 months to 11 years and their caregivers were enrolled in the study, and 367 were screened for adverse events. Recruitment occurred during well-child checks at the University of California San Francisco Benioff’s Children Hospital Oakland (BCH Oakland) Primary Care Clinic, a safety-net practice that provides care to medically underserved or underinsured populations. Eligibility criteria included: not in foster care, English and/or Spanish speaking, had a primary caregiver ≥ 18 years, and not a sibling of an existing study participant. Participation in the larger parent study included four study visits for survey completion (i.e., surveys asked about a plethora of information such as demographics, social needs, physical and mental health, child regulation, and stress), biospecimen collection (i.e., blood, nasal and buccal swabs, and stool), and participation in a social or psychosocial intervention. Participants were compensated up to $300 for their time participating in the entire study (12 months). The 555 participants had a mean age of 5.9 years (standard deviation [SD] = 3.5) and were predominantly male (52.4%), non-Hispanic Black (56.0%), had caregivers with some college education (65.1%), and were low-income. Caregivers were asked about the child’s ACEs using the PEARLS. Responses were collected in item-level screening format (n = 185) (i.e., caregivers disclosed specific adversities their child has experienced), and aggregate-level screening format (n = 182) (i.e., caregivers reported the total number of adversities their child has experienced). Those screened for aggregate-level responses were later asked to provide item-level responses (n = 155, 85.2%). We limited the present analysis to those with item-level responses, yielding a final sample of 340 children. Written informed consent and where appropriate, oral consent, was obtained. The study was approved by the university institutional review board.

### ACEs and related life events

**Fig. 1:**
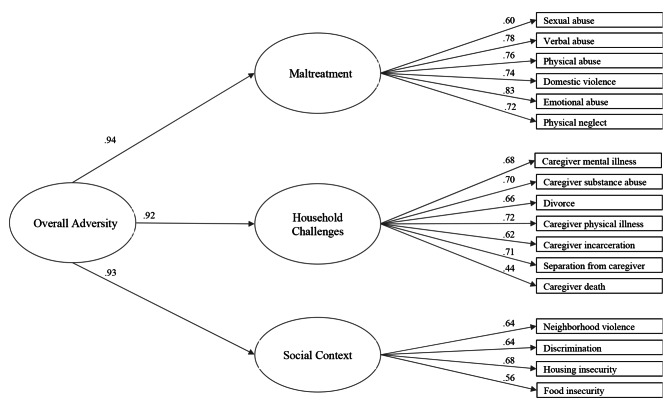
Diagram of Confirmatory Three-Factor Model of the Pediatric ACEs and Related Life Events Screener

ACEs and related life events were measured using the PEARLS, a 17-item pediatric ACEs screen developed for use in clinical practice [21, 22]. The screen included the ten original ACEs categories, plus related life events including children’s lifetime exposure to: discrimination, food insecurity, housing instability, community violence, physical illness/disability of a caregiver, death of a caregiver, and forced separation from caregiver (each item was dichotomized as yes [coded 1] or no [coded 0]) (refer to Fig. 1 for a list of all 17 ACEs and related life events measured). One of the ten ACEs categories includes exposure to domestic violence, which refers to a child witnessing domestic violence.

### Outcomes assessment

We assessed key pediatric clinical, mental, and behavioral health outcomes frequently observed in other ACE literature. Child general health was measured via the Patient-Reported Outcomes Measurement Information System (PROMIS) Global 7-item questionnaire that assessed caregiver-reported physical, mental, and social health, pain, fatigue, and perceived quality of life. Each item response was categorized as excellent (coded 5), very good (coded 4), good (coded 3), fair (coded 2), and poor (coded 1), and the scores were summed across the seven items and analyzed as a continuous variable. Total continuous raw scores were converted into *t*-scores and norm-referenced, in which lower *t*-scores represent poorer general health (*t*-score range 14.7 to 66.1) [23]. We also measured caregiver-reported missed school days due to health (1–9 days [coded 0] vs. ≥10 days [coded 1]) and electronic health record (EHR)-based measures for the 12 months preceding recruitment for emergency department (ED) visits and hospitalizations (any visit [coded 1] vs. none [coded 0]). We assessed child behavioral health as a dichotomous variable using the Behavior Rating Inventory of Executive Function (BRIEF 2/P versions administered to appropriate age group) tool, in which a Global Executive Composite scale *t*-score ≥ 65 was considered clinically significant for poor executive functioning (< 65 [coded 0] vs. ≥ 65 [coded 1]) [24]. Attention Deficit Hyperactivity Disorder (ADHD) diagnosis was based on International Classification of Diseases (ICD)-10 codes in the 12 months prior to recruitment (ADHD coded 1 vs. no ADHD coded 0). Presence of asthma, allergic rhinitis, and atopic dermatitis was obtained using the International Study of Asthma and Allergies in Childhood (ISAAC) questionnaire, which was validated and standardized for international use (presence of atopic disease coded 1 vs. no disease coded 0). Height and weight were obtained from clinical exam, and sex- and age-specific body mass index *z*-scores and percentiles were calculated using 2000 Centers for Disease Control and Prevention growth charts (≥ 95th percentile classified as obese [coded 1] vs. not obese [coded 0]). We used caregiver-reported data on presence of headaches/dizziness and stomachaches in the previous 12 months. EHR ICD-10 codes were retrieved to determine the presence of: (1) acute infections (upper and lower respiratory infection, sinusitis, bronchiolitis, pneumonia, influenza and other viral infections, scarlet fever, otitis media, conjunctivitis, and urinary tract infections); and (2) somatic symptoms (headache, nausea, abdominal pain, epigastric pain, colic, constipation, and migraine) in the 12 months prior to recruitment (presence of conditions [coded 1] vs. no condition [coded 0]).

### Covariates assessment

Socio-demographic covariates were identified a priori based on existing literature on childhood adversities and health outcomes, and they were child age (continuous), sex (male coded as 0 vs. female coded as 1), race/ethnicity (categorized as non-Hispanic White [coded 0], Hispanic [coded 1], non-Hispanic Black [coded 2], and other [coded 3]), caregiver education (categorized as some high school or less [coded 0], high school [coded 1], some college [coded 2], and college [coded 3]), and family income ($25,000 or less coded as 0 vs. greater than $25,000 coded as 1) [25].

### Statistical analysis

Confirmatory factor analysis (CFA) was used to model the latent structure of the PEARLS items using mean- and variance-adjusted weighted least squares estimation (WLSMV) on the tetrachoric correlations of binary PEARLS screen responses [26]. These latent domains are thus derived from the 17 PEARLS items. Standardized factor loadings ≥ 0.40 were used as an inclusion cut-off. One to three-factor models were tested. Model fit was evaluated using indices of absolute fit and standard guidelines of indices, including the model chi-squared statistics, comparative fit index (CFI ≥ 0.95), Tucker Lewis index (TLI ≥ 0.95), and root mean square error of approximation (RMSEA ≤ 0.06) [27–29]. Given the intercorrelation of the PEARLS items and the aggregate and cumulative scoring of ACEs commonly found in the literature [22], a second order factor structure was included in the CFA to represent an overarching summary measure of adversity. Cronbach’s α and McDonald’s ω were used to determine the internal consistency and reliability of the domains. After domains have been determined, composite scores for each PEARLS domain were computed for each participant by summing the number of affirmative responses within a domain.

Next, we estimated a series of nested multiple group CFAs to determine configural (i.e., factor loading pattern and item thresholds are similar and there is overall acceptable model fit in both groups), metric (i.e., factor loadings are constrained to be equal across groups and fits equally as well as the model with the parameters freely estimated), and scalar (i.e., loadings and item thresholds constrained to be equal) measurement invariance by age group (< 6 vs. ≥ 6 years based on school age), gender, and PEARLS screening format (item-level vs. aggregate-level screening). This approach is consistent with the procedure recommended for use with categorical measures outlined by Svetina et al. (2020).

Multivariable logistic and linear regressions were used to examine the relationship between each PEARLS domain and each health outcome, adjusting for covariates. As exploratory analyses, we included all three domains into one model for each of the outcome models to assess collinearity of the domains.

We performed multiple imputation with iterative chained equations to impute missing socio-demographic covariate data (104 participants had missing data for one or more covariates. Missingness across study variables ranged from 1.5 to 30.6%) [31]. Thirty imputed datasets were generated, and we obtained averaged results from the repeated analyses. Participants missing PEARLS or outcome data were excluded from the analyses. The number of missingness for each measured outcome is different (number and percentages of missingness for each outcome are shown in Table 1).

**Table 1:** Child and Caregiver Characteristics

Characteristic		N (%) Total N = 340
Age, mean (SD)		5.8 (3.5)
Sex		
	Male	182 (53.5)
	Female	158 (46.5)
Race		
	Non-Hispanic White	14 (4.1)
	Hispanic	62 (18.2)
	Non-Hispanic Black	188 (55.3)
	Other	76 (22.4)
Caregiver Education		
	Some high school or less	30 (8.8)
	High school	82 (24.1)
	Some college	119 (35.0)
	College	104 (30.6)
	Missing	5 (1.5)
Income		
	25,000 or less	143 (42.1)
	Greater than 25,000	93 (27.4)
	Missing	104 (30.6)
PROMIS *t*-score, mean (SD)		50.5 (8.7)
	Missing	79 (23.2)
Missed School Days Due to Health		
	Less than 10 days	175 (51.5)
	10 days or more	44 (12.9)
	Missing	121 (35.6)
ED visit in the past year		
	No	189 (55.6)
	Yes	151 (44.4)
Hospitalization in the past year		
	No	328 (96.5)
	Yes	12 (3.5)
ADHD		
	No	299 (87.9)
	Yes	41 (12.1)
BRIEF-P Global Executive Composite T score, mean (SD)		55.4 (12.7)
	Missing	148 (43.5)
Stomach Aches		
	No	283 (83.2)
	Yes	46 (13.5)
	Missing	11 (3.2)
Headaches/Dizziness		
	No	293 (86.2)
	Yes	36 (10.6)
	Missing	11 (3.2)
Asthma		
	No	189 (55.6)
	Yes	140 (41.2)
	Missing	11 (3.2)
Rhinitis		
	No	184 (54.1)
	Yes	145 (42.6)
	Missing	11 (3.2)
Eczema		
	No	177 (52.1)
	Yes	151 (44.4)
	Missing	12 (3.5)
Obesity		
	No	257 (75.6)
	Yes	82 (24.1)
	Missing	1 (0.3)
Infections		
	No	174 (51.2)
	Yes	166 (48.8)
Somatic symptoms		
	No	284 (83.5)
	Yes	56 (16.5)
Total ACES score, mean (SD)		3.2 (3.2)

For sensitivity analysis, we compared the averaged results from the multiple imputation to the complete case analysis. Statistical significance was set at *p* ≤ .05. All analyses were performed with R 3.3.2 [32], STATA version 16 [33], SPSS 26 [34], and Mplus 8.5 [35].

## Results

Our study sample (n = 340) was predominantly non-Hispanic Black (n = 188, 55.3%) and low-income (n = 143, 42.1%), with a mean age of 5.8 years (SD = 3.5). Majority of the participants’ caregivers had some college education (n = 223, 65.6%). Participants reported experiencing an average of 3.2 ACEs and related life events (SD = 3.2) (Table 1).

### Confirmatory factor analysis structure

Model fit statistics from the CFA demonstrated that a three-factor model with a second order summary factor adequately fit the data (RMSEA = 0.03; CFI and TLI = 0.99) (Table 2), and exhibited better fit compared to the one and two-factor models (Additional File Table S1). The latent structure is depicted in Fig. 1. Domain 1, labeled as *Maltreatment*, consisted of six items including exposure to sexual abuse, verbal abuse, physical abuse, domestic violence, emotional abuse, and physical neglect (score range 0–6). Exposure to domestic violence was included in the *Maltreatment* domain because literature suggests that a child just witnessing domestic violence has been recognized as a type of child maltreatment [36–38]. Domain 2, labeled *Household Challenges*, consisted of seven items that describe the disturbances in the child’s household: caregiver mental illness, caregiver substance abuse, divorce or separation, caregiver physical illness, caregiver incarceration, death of caregiver, and child separation from caregiver (score range 0–7). Domain 3, labeled *Social Context*, captures four items in the PEARLS that may influence the child’s social environment: neighborhood violence and whether the child experienced discrimination, housing insecurity, or food insecurity (score range 0–4).

**Table 2:** Factor Loadings and Scale Reliability Estimates for the Second Order CFA Model

				Loading*	Reliability	
**Factor**	**Indicator**		**% Exposed**	β	ω	ɑ
*First-order*						
Maltreatment	Sexual Abuse		4	0.60	0.73	0.87
	Verbal Abuse		14	0.78		
	Physical Abuse		6	0.76		
	Domestic Violence		34	0.74		
	Emotional Abuse		18	0.83		
	Physical Neglect		9	0.72		
Household Challenges	Mental Illness		39	0.68	0.70	0.82
	Substance Abuse		20	0.70		
	Divorce		30	0.66		
	Physical Illness		14	0.72		
	Incarceration		23	0.62		
	Separation from Caregiver		11	0.71		
	Caregiver Death		9	0.44		
Social Context	Neighborhood Violence		24	0.64	0.55	0.70
	Discrimination		15	0.64		
	Housing Insecurity		24	0.68		
	Food Insecurity		19	0.56		
* Second-order*						
Overall Adversity	Maltreatment			0.94		
	Household Challenges			0.92		
	Social Context			0.93		
Model fit indices: *Χ*^2^(116) = 139.68, *p* = .07; RMSEA = 0.03 [90% CI: 0.00, 0.04]; CFI = 0.99; TLI = 0.99						

*All loadings were statistically significant, *p* < .05

Correlations among the three domains ranged from 0.85 to 0.88. The subscales had acceptable internal consistency and reliability, with alphas ranging from 0.70 to 0.87 and omegas from 0.55 to 0.73 (Table 2). The average number of exposures for each domain was as follows: 0.8 for *Maltreatment* (SD = 1.2), 1.5 for *Household Challenges* (SD = 1.6), and 0.8 for *Social Context* (SD = 1.0).

### Measurement invariance for age, gender, and screening format

A summary of the fit statistics for the multiple group CFAs for age group, gender, and PEARLS screening format is shown in Table 3. Configural invariance was established for age, gender, and screening format. Metric invariance was substantiated for gender and screening format, but not for age group. There was a decrease in the mean scores across all three domains for 5-year-olds (Additional File Figure S1). Finally, we observed scalar invariance for gender and screening format when comparing the model with constrained loadings and item thresholds to the previous less constrained model (Table 3).

**Table 3:** Summary of Fit Statistics of Measurement Invariance Models across Age, Gender, and PEARLS Screening Format

		Chi-square	Degrees of freedom	*p*-value^a^	RMSEA	CFI	TLI
Child age (< 6 v 6 + years)							
	Configural	268.26	232	--	0.03	0.96	0.96
	Constrain thresholds	393.42	249	< 0.001	0.06	0.85	0.83
	Constrain thresholds + loadings	399.02	266	0.05	0.05	0.86	0.86
Screening format							
	Configural	285.10	232	--	0.04	0.96	0.95
	Constrain threshold	309.65	249	0.07	0.04	0.95	0.95
	Constrain thresholds + loadings	321.08	266	0.25	0.04	0.96	0.96
Gender							
	Configural	289.16	232	--	0.04	0.95	0.94
	Constrain threshold	299.99	249	0.76	0.04	0.96	0.95
	Constrain thresholds + loadings	321.08	266	0.25	0.04	0.96	0.96

^a^Comparison between one measurement invariance model to the one above

### Associations between PEARLS domains and child outcomes

After adjusting for covariates, we found statistically significant associations between the three domains and child general, mental, and physical health outcomes. All three PEARLS domains when modelled individually had a statistically significant association with lower caregiver ratings of child’s general health as assessed by PROMIS and clinically poorer Global Executive Functioning as measured by BRIEF (Table 4). For specific health outcomes, there were increased odds of stomachaches with greater exposure to *Maltreatment*, *Household Challenges*, and adverse *Social Context* factors. The *Social Context* domain was additionally associated with headaches (odds ratio [OR] = 1.55; 95% confidence interval [95% CI] [1.10, 2.18]) and somatic symptoms (OR = 1.40; 95% CI [1.05, 1.86]). There was a positive association between eczema and both the *Maltreatment* (OR = 1.24; 95% CI [1.02, 1.52]) and *SocialContext* domains (OR = 1.49; 95% CI [1.17, 1.89]) (Table 4).

**Table 4:** Associations between PEARLS Factors Modelled Individually and Child Health

Health outcomes	Maltreatment OR (95% CI)^a^	Household challenges OR (95% CI)^a^	Social context OR (95% CI)^a^
PROMIS^b^	**-2.29 (-3.14, -1.44)**	**-1.51 (-2.15, -0.87)**	**-2.32 (-3.30, -1.33)**
Missed school days	1.19 (0.91, 1.56)	1.21 (0.98, 1.49)	1.33 (0.97, 1.83)
ED visits	0.85 (0.70, 1.04)	0.96 (0.83, 1.11)	1.07 (0.86, 1.34)
Hospitalization	0.61 (0.28, 1.32)	1.06 (0.72, 1.57)	0.62 (0.29, 1.34)
ADHD	1.17 (0.89, 1.52)	1.20 (0.98, 1.48)	1.09 (0.77, 1.53)
BRIEF	**2.48 (1.75, 3.51)**	**1.59 (1.26, 2.01)**	**2.35 (1.64, 3.37)**
Stomachaches	**1.38 (1.08, 1.78)**	**1.31 (1.08, 1.59)**	**1.45 (1.07, 1.96)**
Headaches	1.24 (0.94, 1.64)	1.15 (0.93, 1.42)	**1.55 (1.10, 2.18)**
Asthma	1.07 (0.86, 1.33)	1.16 (0.99, 1.37)	1.21 (0.94, 1.57)
Rhinitis	1.15 (0.93, 1.42)	1.13 (0.97, 1.32)	1.21 (0.95, 1.55)
Eczema	**1.24 (1.02, 1.52)**	1.10 (0.95, 1.27)	**1.49 (1.17, 1.89)**
Obesity	0.98 (0.79, 1.22)	0.99 (0.84, 1.17)	1.09 (0.84, 1.40)
Infections	0.85 (0.70, 1.04)	0.97 (0.84, 1.13)	1.02 (0.81, 1.29)
Somatic symptoms	1.01 (0.78, 1.31)	1.04 (0.85, 1.26)	**1.40 (1.05, 1.86)**

^a^Models adjusted for child’s age, sex, race/ethnicity, caregiver’s educational level, family income, and screening format. Race/ethnicity was categorized as Non-Hispanic White, Non-Hispanic Black, Hispanic, or other; caregiver’s educational level was categorized as some high school or less, high school graduate, some college, college or greater; family income was dichotomized as <$25,000 vs. ≥ $25,000 annually based on the sample distribution and approximation of federal poverty level for a family of four

^b^Results are mean differences (95% CI)

When all three domains were included simultaneously in a model, some associations with child health were attenuated. Only *Maltreatment* remained significantly associated with PROMIS, and both *Maltreatment* and *Social Context* were independently associated with poorer Global Executive Functioning. Only *Social Context* remained significantly associated with physical health symptoms of headaches, eczema, and somatic symptoms (Additional File Table S2).

In sensitivity analyses, the pattern of results from the complete case and multiple imputation analyses was virtually identical.

## Discussion

In this psychometric analysis of the PEARLS used in a pediatric population of a safety-net primary care clinic, the findings revealed a three-domain structure with *Maltreatment*, *Household Challenges*, and *Social Context* dimensions as well as a higher order general domain of cumulative child adversity. Findings also demonstrated measurement equivalence across both gender and PEARLS screening format. All three domains were associated with poorer general and behavioral health as well as with stomachaches. Both the *Maltreatment* and *Social Context* domains were associated with eczema, while the *Social Context* domain was additionally associated with headaches. Findings support the presence of three PEARLS domains with practical implications for resource linkage and intervention and demonstrate that each unique relational pattern in the PEARLS items may have an impact on health outcomes. Our validation of the three distinct domains offers a more psychometrically robust approach that is content-specific with immediate clinical applicability to strengthen tailored responses including better identification and distribution of subsequent resources in the clinical, research, and policy settings for exposed individuals that align with each domain.

Findings for the PEARLS domains are consistent with related literature to date [39]. Other studies have similarly derived *Maltreatment* and *Household Challenges* subdomains within the traditional ACE categories [1, 40]. Using confirmatory factor analysis, a Canadian study of adolescents found that child maltreatment and household challenges domains were associated with poor mental and physical health, but did not identify a social-environmental factor [41]. A birth cohort study in the United Kingdom derived a “socioeconomic and material disadvantage” (16 p396) domain from the National Survey of Health and Development and found that it was associated with poorer general health in later life [16], similar to our finding with the *Social Context* domain and outcomes. Although no other study has assessed the same three subdomains of *Maltreatment*, *Household Challenges*, and *Social Context* as latent constructs, one study of court-involved youth looked at three similar theory-derived domains of maltreatment, family dysfunction, and social disadvantage and found similar increased risks for mental health problems with maltreatment and household dysfunction [42].

Findings from the current study additionally support PEARLS domain measurement invariance, which has been largely left out of most of the previous assessments of childhood adversity constructs [11, 13–17, 41, 43–45]. Establishing measurement invariance allows more confident ascertainment of true group differences in a given construct instead of differences based on measurement bias or differences in measure interpretation [19]. We established measurement invariance across both gender and screening format, suggesting that meaningful comparison of domain scores can be made across males and females and across item-level and aggregate-level response ACE screening. We were not able to demonstrate measurement invariance across age groups. There are several possible reasons for this finding. First, a pattern was present in the mean scores across all three domains, in which there was a dip in all domain scores for 5-year-olds, suggesting there may not be static changes in these domains with development. Furthermore, we chose age six as an arbitrary but meaningful cut-point based on school-age and choosing another cut-point might show different results. Whether the PEARLS subscales should be interpreted differently for these age groups requires further investigation that is outside the scope of this analysis.

While all three domains were associated with child health outcomes, our exploratory analyses in which all three domains were included in one model suggest specific independent associations. All three domains were associated with PROMIS score as demonstrated by the three separate models, with *Maltreatment* capturing additional variance that is not measured by *Household Challenges* nor *Social Context* as shown by the exploratory model, as statistical significance was only retained for *Maltreatment*. We also observed this with eczema, in which statistical significance was only retained for *Social Context*. This pattern of results is not unexpected given the collinearity between the domains and adversities. The observed pattern of relatedness among the domains suggests that a higher-order overall PEARLS domain exists, which we accounted for using a second order model, and have previously reported on the cumulative risk association with health outcomes [22]. While all three PEARLS domains may be statistically related to some outcomes, suggesting that a broad set of adversities may account for the associations, other outcomes may be associated with fewer domains with different patterns of associations, suggesting that a more specific set of adversities may play a role. Given the diversity of outcomes found to be associated with ACEs, screening across all domains may improve the detection of a child at risk for poor health outcomes. Future research should build evidence on which interventions or resources best support each domain and develop clinical workflows that facilitate support for children and families exposed to each domain.

Establishing domains within the PEARLS as predictors of various health outcomes has immediate implications for clinical practice and policymaking. First, distinct domains provide the opportunity for targeted and tailored treatment and linkage to referrals and resources. Limited accessible supports for children and families who experience adversity and following up positive endorsements of adversities with appropriate, specific interventions and resources are two of the biggest barriers to clinicians supporting trauma screening [46, 47]. If supports can be targeted based on latent domains, more tailored resource and referral linkages can be provided thus maximizing use of often limited resources while avoiding overwhelming services with well-meaning but mis-targeted referrals. Second, clinicians may be less likely to miss or overlook resource linkage opportunities among children with relatively lower cumulative scores if they observe that the few items that are endorsed all fall within one domain. Third, domain scores may also enhance screening by streamlining clinical workflows, while acknowledging that the specific resource within that workflow will depend on the specific adversity. Domains may help point to follow-up screening required for current risk. For example, after administering the initial PEARLS, if clinicians find that their patients endorse one or multiple items within a single domain, they may further investigate the patients’ exposure to similar adversities as part of a more focused follow-up. Use of the distinct domains in this manner may help tailor the response and expedite referral and access to resources. For example, in addition to clinician involvement and anticipatory guidance and follow-up for any ACE exposure, if a patient has a high score for *Maltreatment*, a clinician may directly refer to an in-house social worker, child protective services (CPS), or mental health resources.

There are some limitations to consider when interpreting these results. First, our study, similar to many other ACEs studies, relied on caregiver-report. Some questions, such as those related to child abuse, may be sensitive and anxiety-inducing to report, potentially leading to underreporting of ACEs [48]. This may also lead to common method variance bias, which occurs when there is variance due to measurement method rather than to the constructs the measures are assumed to represent. However, the observed screening format invariance provides some evidence that differences observed are not due to measurement mode. Also, generalizability of the results is limited because this study focused on pediatric patients in an urban, primary care center that cares for mostly underserved families; thus, further validation is needed in more diverse populations with other age groups and settings. However, these findings can still serve as preliminary construct validation of the PEARLS. In addition, because of the small sample size, our study may be underpowered to detect significant differences. Participants with missing PEARLS or outcome data were excluded from the study, limiting the sample size, and these participants may be different from those who had this data. We also had a large percentage of missingness for the income variable, which we addressed with multiple imputation and the results between the complete case and multiple imputation analyses were similar. Lastly, the timing of certain measurements may have also impacted our results, specifically the EHR data that was collected 12 months prior to recruitment. Some of the younger children may not have as much time to have codes for certain health outcomes reported in the EHR or may not be able to express their exact condition compared to older children, but we attempted to address this by adjusting for age. Also, the PEARLS assesses lifetime prevalence of adversities rather than for exposures that occur at specific time-points after recruitment. Given the cross-sectional design of the analysis, causation cannot be determined. As such, future studies should focus on the longitudinal effects of these adversity domains on health outcomes and how timing and duration of adversity exposure can also impact disease risk. Future studies should also assess how providing tailored responses based on domain positivity moves us toward patient-centered care and helps prevent poor health outcomes.

## Conclusion

As a prevention tool, PEARLS screening early in childhood offers an opportunity to link families to resources prior to the onset of negative health outcomes and to mitigate and decrease exposures to childhood adversity. The PEARLS is an efficient way to cumulatively assess *Maltreatment*, *Household Challenges*, and *Social Context* domains while also tailor referral and intervention needs based on domain positivity. All three domains were associated with poorer health in children and complement the current practice of assessing cumulative risk of childhood adversity which can help guide clinical practice and policy.

## Electronic supplementary material

Below is the link to the electronic supplementary material.

Supplementary Material 1

## Data Availability

The de-identified datasets generated and/or analyzed during the current study are available from the corresponding author on reasonable request and on signing of a data-sharing agreement.

## Abbreviations

ACEs: Adverse Childhood Experiences
PEARLS: Pediatric ACEs and Related Life Events Screener
BCH Oakland: Benioff’s Children Hospital Oakland
PROMIS: Patient-Reported Outcomes Measurement Information System
EHR: Electronic health record
ED: Emergency department
BRIEF: Behavior Rating Inventory of Executive Function
ADHD: Attention Deficit Hyperactivity Disorder
ICD: International Classification of Diseases
ISAAC: International Study of Asthma and Allergies in Childhood
CFA: Confirmatory factor analysis
WLSMV: Mean- and variance-adjusted weighted least squares estimation
CFI: Comparative fit index
TLI: Tucker Lewis index
RMSEA: Root mean square error of approximation
SD: Standard deviation
OR: Odds ratio
CI: confidence interval
CPS: Child protective services
