# Supplementary material for: Pediatric ACEs and related life event screener (PEARLS) latent domains and child health in a safety-net primary care practice
Source: BMC Pediatr. 2023 Jul 17;23:367. doi: 10.1186/s12887-023-04163-2 (PMC10351141; doi:10.1186/s12887-023-04163-2)
Supplement: Supplementary file 1 — Supplementary Material 1 [file 12887_2023_4163_MOESM1_ESM.docx]

**Supplement**

**Figure S1.** Domain mean scores across age

**
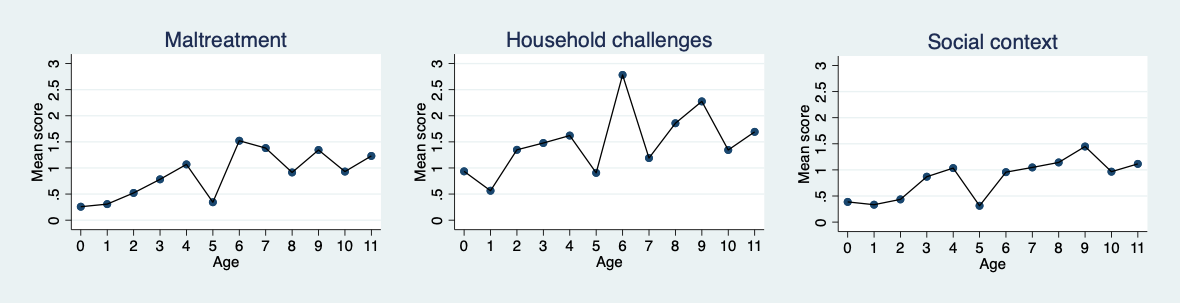
**

**Table S1.** Fit Statistics for One to Three Factor CFA Models

|  |  | Chi-square | Degrees of freedom | *p*-value^a^ | RMSEA | CFI | TLI |
| --- | --- | --- | --- | --- | --- | --- | --- |
| One factor | | 151.334 | 119 | - | 0.028 | 0.987 | 0.985 |
|  |  |  |  |  |  |  |  |
| Two factors | | 148.461 | 118 | 0.06 | 0.028 | 0.988 | 0.986 |
|  |  |  |  |  |  |  |  |
| Three factors | | 139.679 | 116 | 0.02 | 0.025 | 0.990 | 0.989 |

^a^ Comparison between one model to the one above

**Table S2.** Associations between PEARLS factors and child health with all domains in one model

| **Health outcomes** | **Maltreatment OR (95% CI)^a^** | **Household challenges OR (95% CI)^a^** | **Social context OR (95% CI)^a^** |
| --- | --- | --- | --- |
| PROMIS^b^ | **-1.36 (-2.45, -0.28)** | -0.59 (-1.38, 0.21) | -1.07 (-2.24, 0.11) |
| Missed school days | 1.01 (0.72, 1.42) | 1.13 (0.87, 1.46) | 1.22 (0.84, 1.77) |
| ED visits | 0.78 (0.61, 1.00) | 0.99 (0.83, 1.18) | 1.24 (0.94, 1.62) |
| Hospitalization | 0.55 (0.22, 1.42) | 1.47 (0.90, 2.41) | 0.60 (0.24, 1.49) |
| ADHD | 1.05 (0.75, 1.47) | 1.21 (0.93, 1.56) | 0.92 (0.61, 1.38) |
| BRIEF | **1.96 (1.31, 2.93)** | 1.08 (0.80, 1.45) | **1.65 (1.10, 2.49)** |
| Stomachaches | 1.17 (0.86, 1.59) | 1.17 (0.92, 1.49) | 1.19 (0.83, 1.71) |
| Headaches | 1.10 (0.77, 1.57) | 0.97 (0.74, 1.29) | **1.51 (1.02, 2.24)** |
| Asthma | 0.91 (0.70, 1.20) | 1.16 (0.94, 1.42) | 1.14 (0.84, 1.56) |
| Rhinitis | 1.04 (0.80, 1.35) | 1.07 (0.88, 1.30) | 1.13 (0.84, 1.51) |
| Eczema | 1.12 (0.87, 1.44) | 0.94 (0.78, 1.13) | **1.46 (1.10, 1.94)** |
| Obesity | 0.95 (0.72, 1.25) | 0.97 (0.79, 1.19) | 1.15 (0.85, 1.55) |
| Infections | 0.79 (0.61, 1.01) | 1.03 (0.86, 1.23) | 1.15 (0.87, 1.51) |
| Somatic symptoms | 0.86 (0.62, 1.19) | 0.95 (0.75, 1.21) | **1.57 (1.11, 2.21)** |

^a^Models adjusted for child’s age, sex, race/ethnicity, caregiver’s educational level, family income, screening arm, and the other two domains

^b^Results are mean differences (95% CI)
